# The Impact of Lignin Biopolymer Sources, Isolation, and Size Reduction from the Macro- to Nanoscale on the Performances of Next-Generation Sunscreen

**DOI:** 10.3390/polym16131901

**Published:** 2024-07-02

**Authors:** Victor Girard, Léane Fragnières, Hubert Chapuis, Nicolas Brosse, Laurent Marchal-Heussler, Nadia Canilho, Stéphane Parant, Isabelle Ziegler-Devin

**Affiliations:** 1 Laboratoire d’Etude et de Recherche sur le MAtériau Bois (LERMAB), Faculty of Science and Technology, University of Lorraine, F-54000 Nancy, France; leane.fragnieres@etu.unistra.fr (L.F.); hubert.chapuis@univ-lorraine.fr (H.C.); nicolas.brosse@univ-lorraine.fr (N.B.); isabelle.ziegler@univ-lorraine.fr (I.Z.-D.); 2 Ecole Nationale Supérieure des Industries Chimique (ENSIC), University of Lorraine, F-54000 Nancy, France; laurent.marchal-heussler@univ-lorraine.fr; 3 Laboratoire Lorrain de Chimie Moléculaire (L2CM), Faculty of Science and Technology, University of Lorraine, F-54000 Nancy, France; nadia.canilho@univ-lorraine.fr (N.C.); stephane.parant@univ-lorraine.fr (S.P.)

**Keywords:** lignin, sunscreens, lignin nanoparticles, organosolv, UV shielding, eco-friendly production

## Abstract

In recent years, concerns about the harmful effects of synthetic UV filters on the environment have highlighted the need for natural sun blockers. Lignin, the most abundant aromatic renewable biopolymer on Earth, is a promising candidate for next-generation sunscreen due to its inherent UV absorbance and its green, biodegradable, and biocompatible properties. Lignin’s limitations, such as its dark color and poor dispersity, can be overcome by reducing particle size to the nanoscale, enhancing UV protection and formulation. In this study, 100–200 nm lignin nanoparticles (LNPs) were prepared from various biomass by-products (hardwood, softwood, and herbaceous material) using an eco-friendly anti-solvent precipitation method. Pure lignin macroparticles (LMPs) were extracted from beech, spruce, and wheat straw using an ethanol–organosolv treatment and compared with sulfur-rich kraft lignin (KL). Sunscreen lotions made from these LMPs and LNPs at various concentrations demonstrated novel UV-shielding properties based on biomass source and particle size. The results showed that transitioning from the macro- to nanoscale increased the sun protection factor (SPF) by at least 2.5 times, with the best results improving the SPF from 7.5 to 42 for wheat straw LMPs and LNPs at 5 wt%. This study underscores lignin’s potential in developing high-quality green sunscreens, aligning with green chemistry principles.

## 1. Introduction

In a world where the growing concerns of environmental sustainability and improved ultraviolet (UV) radiation levels caused by ozone layer depletion due to human activity converge, the development of sunscreens that are both eco-friendly and effective in shielding against harmful UV radiation has become an increasingly pressing challenge.

Sunburn, skin damage, or the potential development of cancer can be attributed to prolonged UVB (290–320 nm) exposure compared to less energetic UVA (320–400 nm) [1,2]. In response to these concerns, traditional commercial sunscreen has relied on contentious organic and inorganic UV filters [3], such as titanium dioxide [4] (TiO_2_), to protect human skin from UV rays despite significant apprehensions, including high environmental impact and persistence [5], skin sensitivities, and limited UV protection spectrum [6]. As an example, it has recently been proven that common chemical or physical UV absorbers, such as ZnO, TiO_2_, oxybenzone, octocrylene, or octinoxate, are responsible for marine ecosystem modification [7] and coral bleaching [8,9]. This prompted Australia and Hawaii [7,10] to prohibit these UV-active components with respect to the annual 14,000 tons [11] of sunscreen that end up in the world’s oceans [12].

**Figure 1:**
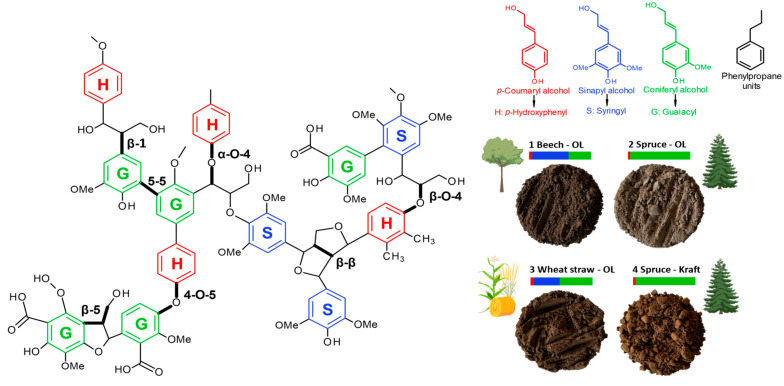
Lignin chemical structure with the main linkages and the proportion of different monolignols and monomeric units (G, S, and H) according to the biomass source. Photographs of the organosolv (OL)-extracted lignin’s with beech (1), spruce (2), and wheat straw (3). Comparison with kraft lignin (KL) from spruce (4).

In this context, lignin—the most abundant aromatic biopolymer [13,14,15,16] constituting 30% of the non-fossil organic carbon in nature [17,18] with proven high UV absorption [19,20,21,22,23,24,25,26,27,28,29,30,31] and low cytotoxicity [32,33,34,35,36,37]—presents tremendous potential for next-generation sunscreens. Lignin’s complex polymer structure contains phenylpropane units composed of guaiacyl (G), syringyl (S), and p-hydroxypenyl (H) monomeric units linked by aryl ether and carbon–carbon bonds (Figure 1) [38,39]. Furthermore, lignin’s UV-absorbing properties are related to functional groups, such as phenolic, quinones, and methoxy substituted groups, and other chromophores [40,41,42,43], making this sustainable polymer an excellent absorber in the UVB–UVA wavelength areas targeted by a wide range of sunscreens [12].

Annually, 50 million tons of generated lignin is used as an energy source [44,45,46]. Despite its great potential, lignin is seldom used for non-energy applications due to processing challenges [47], the inherent heterogeneity of the macromolecular structure depending on isolation methods and biomass nature [48], and particles that are too large, limiting industrial applications. Currently, lignin studies and valorizations are mainly focused on technical lignin materials [49], such as lignosulfonate or kraft lignin (KL), regardless of environmental limitations [50,51] and the presence of sulfur [17,52], which immediately hinders many possible uses, such as cosmetics [53].

However, lignin biopolymers could take advantage of novel eco-friendly isolation processes, similar to organosolv [51], and they can also take advantage of the emerging integration of nanotechnology in cosmetics to address current limitations. Precisely, the extensive reach of nanotechnology in the cosmetic industry, as observed in inorganic ZnO and TiO_2_ UV filters, results from the enhanced properties of nanoparticles, including their size, stability, shape, reactivity, color, or solubility [54]. Recent studies showed that lignin’s inherent heterogeneity, dark color, and poor dispersibility could be overcome thanks to particle size reduction [26], while maintaining a size of over 45 nm to avoid skin absorption risks [55] and meet the Scientific Committee on Consumer Safety (SCCS) marketing requirements. For example, Qian et al. [22] showed that the sun protection factor (SPF) of mixed sunscreens with lignin is boosted according to particle size reduction.

Various methods, such as nano-precipitation [56,57,58,59,60,61,62], mechanical and ultrasound treatments [63,64,65,66,67], or aerosol processing [68], have been investigated for producing lignin nanoparticles (LNPs) with linked properties (size, shape, stability, and reactivity) [69]. However, previous studies predominantly used kraft lignin, without consistently specifying the biomass source or thoroughly examining the production yields and the actual impact of particle size reduction on UV-absorbing properties. This limitation results in an incomplete understanding of the value of lignin potential for next-generation sunscreen.

Knowing that the organosolv isolation process produced higher UV-shielding lignin compared to other extraction methods, as demonstrated by Qian et al. [20] and Tan et al. [70], this study employed, for the first time, a global sustainable method from various biomass by-products using only ethanol, water, and heat for pure lignin macroparticles (LMPs) extraction via organosolv pretreatment (OL) and particle size reduction (nano-precipitation). This new top-down lignin-based approach, described for the first time by Girard et al. [71], allowed the production of different eco-friendly concentrated LNPs suspensions with improved characteristics while having high production yields. Then, LMPs and LNPs-based formulations were developed to highlight the size reduction in UV-absorbing properties with respect to used pure cream and sunscreen. As lignin’s UV-absorbing properties are related to its chemical structure (assessed via SEC and HSQC and ^31^P NMR), which varies according to the feedstock source, the prepared sunscreens with different inputs (hardwood, softwood, and grasses) and lignin types (KL and OL) were analyzed to determine the most suitable lignin chemical structure for UV absorption. The performance of lignin-based formulations was compared to that of commercial SPF 10 and SPF 30 sunscreens containing TiO_2_ nanoparticles in order to assess the commercialization potential of lignin-based formulations.

## 2. Materials and Methods

### 2.1. Raw Materials

Commercial kraft lignin was provided by the Lineo^™^ Prime W by Stora Enso process (CAS Number 8068-05-1, Kotka, Finland). Organosolv (OL) lignin was extracted from three distinct and local biomass by-products from Vosges forests and meadows (Epinal, France). Selected by-products included beech (*Fagus sylvatica*), spruce (*Picea abies* L.), and wheat straw (*Triticum*) provided by the French National School of Wood Technologies and Timber Engineering (ENSTIB, Épinal, France) and the “Bergerie de Straiture” sheepfold (Ban-Sur-Meurthe-Clefcy, France). NIVEA^®^ Soft moisturizing skin care cream (200 mL, Hambourg, Germany) (Art.-No. 89050) was used as the basis for the formulation of sunscreen; this cream is mentioned as “pure cream” hereafter. Sunscreen lotion comprised GARNIER^®^ Latte protettivo SPF 10 (200 mL, Loudéac, France) and CIEN^®^ Sun SPF 30 (75 mL, Paris, France). Cream and sunscreen were purchased from European drug markets.

### 2.2. Biomass Chemical Characterization

Chemical composition analysis, following the National Renewable Energy Laboratory (NREL) labeling protocols and TAPPI method T222, was conducted on biomass powder. The procedure involved ash content determination (NREL/TP-510-42622), Soxhlet extraction (NREL/TP-510-42619), and analyses of acid-insoluble lignin and monomeric sugar contents from free extractive biomass (NREL/TP-510-42618 and TP-510-42623). High-performance anion exchange chromatography coupled with pulsed amperometry detection (HPAE-PAD, ICS-3000 Dionex^®^, Sunnyvale, CA, USA) was employed for monomeric sugar analyses (CarboPac PA-20 Dionex^®^ (Waltham, MA, USA) analytical column). The composition’s gradient involved ultrapure water and 250 mM NaOH solutions eluted at 35 °C and 0.4 mL/min. Fucose, arabinose, rhamnose, galactose, glucose, xylose, mannose, galacturonic acid, and glucuronic acid were determined via external calibration (Sigma-Aldrich, St Louis, MI, USA). Chemical content comprised the following: (1) beech: extractives = 2.67 ± 0.27 (%, *w*/*w*), cellulose = 47.80 ± 1.47 (%, *w*/*w*), hemicelluloses = 22.52 ± 0.86 (%, *w*/*w*), lignin = 23.70 ± 0.25 (%, *w*/*w*), and ashes 0.73 ± 0.01 (%, *w*/*w*); (2) spruce: extractives = 2.14 ± 0.24 (%, *w*/*w*), cellulose = 45.06 ± 1.19 (%, *w*/*w*), hemicelluloses = 21.41 ± 1.19 (%, *w*/*w*), lignin = 27.86 ± 0.33 (%, *w*/*w*), and ashes 0.36 ± 0.01 (%, *w*/*w*); (3) wheat straw: extractives = 0.56 ± 0.15 (%, *w*/*w*), cellulose = 49.56 ± 1.05 (%, *w*/*w*), hemicelluloses = 23.21 ± 0.69 (%, *w*/*w*), lignin = 20.52 ± 0.24 (%, *w*/*w*), and ashes 6.34 ± 0.07 (%, *w*/*w*).

### 2.3. Lignin Macroparticles (LMPs) Extraction

Macroscale lignin from local biomass was obtained by employing OL treatment. For this purpose, biomass was first coarsely crushed into ø8 mm particles with a Retsch^®^ cross beater mill SK100 (Düsseldorf, Germany) prior to the OL extraction process. In a pressurized PARR^®^ 4568 2-L benchtop reactor (Moline, Illinois, United States), 100 g of dry biomass underwent 1 h treatment at 200 °C in a 60/40 *v*/*v* EtOH/H_2_O solution, maintaining a liquid–solid ratio of 10/1. The reactor was then quickly cooled in an ice bath to stop the chemical reaction. Extracted black liquor was separated from the solid phase through vacuum filtration. The lignin contained in the black liquor mixture was isolated via direct precipitation with cold distilled water at a 1/3 *v*/*v* ratio. After 1 h, the precipitated lignin was filtered via vacuum filtration using a 1.6 µm glass macrofibre filter. Finally, the separated lignin was washed with 500 mL of distilled water before drying in a controlled oven at 40 °C for 2 days. LMPs powder was stored in a dark room before further analyses. The milled wood lignin (MWL) extraction method is based on preliminary findings from our research group [72].

### 2.4. Lignin Nanoparticles (LNPs) Preparation

LNPs were synthesized through a simple, environmentally friendly anti-solvent precipitation method. Initially, each LMPs fraction was dissolved in an 80% *v*/*v* EtOH/H_2_O solution with ultrasonic treatment for 1 h to ensure complete lignin solubilization at a concentration of 30 mg/mL. The resulting solution sustained vacuum filtration using a 0.45 μm nylon filter to eliminate potential aggregates, which represented between 0.9 and 3.1% of the total solubilized mass for beech, spruce, and wheat straw compared with 6.8% for KL. The production of LNPs is performed thanks to the addition of the LMPs solution to the anti-solvent phase with a KF Technology^®^ NE-1010 (Roma, Italy) syringe pump. Each phase is kept at 20 °C, and the addition of the LMPs phase is performed under magnetic stirring (500 rpm) at a constant flow rate of 100 mL/min until achieving a final concentration of 3 mg/mL. The resulting LNPs suspensions were predominantly aqueous, with a composition of 91/9 H_2_O/EtOH *v*/*v*. Suspensions were stored at 4 °C and further freeze-dried for 48 h using a BILON^®^ FD-1A-50 freeze dryer (Beijing, China) to obtain dried LNPs.

### 2.5. Elemental Analysis

Thermo Finnigan Flash EA^®^ 112 Series (Waltham, MA, USA) was used to perform sulfur elemental analysis. Sample combustion (1.5 mg) was performed for 15 s at a high temperature (1000 °C) under an oxidizing atmosphere and in the presence of tungstic anhydride. Produced gaseous products (H_2_O, SO_2_, CO_2_, and NOx) were reduced to N_2_ in the presence of copper; then, they were analyzed via gas chromatography. The percentage of sulfur present in the compound was calculated by using Eager 300 software.

### 2.6. Size Exclusion Chromatography (SEC)

SEC was applied to analyze LMPs fractions for molecular weight distributions and averages. Each lignin sample, initially dissolved in 10 mM NaOH at 5 mg/mL under 24 h magnetic agitation, was filtered with 0.45 μm PTFE filters. Shimadzu Prominence^™^ (Nara, Japan) chromatography outfitted with a Shimadzu SPD-20A UV detector (280 and 254 nm), a refractive index detector (RID, Shimadzu RID-20A), and Shodex^™^ GPC KF-806L (Munich, Germany) and Phenogel^™^ 00H-0442-K0 (Torrance, CA, USA) columns were applied. The separation occurred at 35 °C, with elution using 10 mM NaOH at a flow rate of 0.4 mL/min. The calibration curve was plotted using Aligent Technologies^®^ GPC/SEC calibration kits (Aligent PL2090-0101, Santa Clara, CA, USA) and the Steinmetz et al. [73] method.

### 2.7. Nuclear Magnetic Resonance (NMR)

The examination of the lignin structure involved both heteronuclear single quantum coherence (HSQC) and ^31^P NMR. In a concise procedure for HSQC, 100 mg of purified and dried LMPs was dissolved in 700 μL of dimethyl sulfoxide-d6 (DMSO-d6 99.8%). Spectra were acquired using the Bruker^®^ Avance III 400 MHz spectrometer (Billerica, Massachusetts, United States) at 50 °C with a relaxation delay of 1.5 s. For ^31^P NMR, the hydroxyl group’s content was determined following the published methodology [74], where 25 mg of purified LMPs was dissolved in 400 μL of pyridine/deuterated chloroform (1.6/1 *v*/*v*). A mixed solution (A) of chromium (III) acetylacetonate 97% (3.6 mg/mL) and cyclohexanol (4.0 mg/mL of A) served as the relaxation reagent and internal standard. The solution was derivatized with 50 μL of 2-chloro-4,4,5,5-tetramethyl-1,3,2-dioxaphospholane (TMDP), vortexed, and analyzed using the Bruker^®^ Avance III HD 300 MHz spectrometer at 25 °C with a 25 s relaxation delay. NMR data were processed using Topspin^®^ 4.1.0 software (Bruker Bio Spin, Billerica, MA, USA).

### 2.8. Dynamic Light Scattering (DLS)

The Malvern^™^ Zetasizer ULTRA instrument (Grovewood, UK) was used to determine the size distribution, size average, polydispersity index (PDI), and ζ-potential of the prepared LNPs suspensions. LNPs suspensions were analyzed immediately after precipitation at 25 °C via complete optical PS cells with a volume of 1.5 mL. Triplicate measurements were recorded in the DLS mode at an angle of 174°. ζ-potential analyses were performed using the same instrument, employing special folded capillary Zeta cells (DTS 1070) at 25 °C.

### 2.9. Transmission Electron Microscopy (TEM)

LNPs images were captured through an FEI Philips^®^ CM200 transmission electron microscope (TEM, Amsterdam, The Netherlands), operating at an accelerating voltage of 160 kV. The samples were directly prepared by applying a drop of LNPs suspension (3 mg/mL) without contrasting agents onto a TEM grid and dried for 30 min.

### 2.10. Lignin-Based Sunscreen Preparation and Study

As in previous studies [19,27,75], the preparation of sunscreens and the associated SPF measurements were conducted as follows: Each lignin-based sunscreen formulation was generated by mixing LMPs or LNPs powder with corresponding sunscreen or pure cream at 1, 5, and 10 wt% using an IKA^®^ magnetic stirrer at 400 rpm for 14 h in a dark room. After blending, 37.5 mg of lignin-based sunscreen was applied onto a clean 7.5 × 2.5 cm quartz plate of 2 mm thickness to comply with the International sun protection factor (SPF) test method conditions of 2.0 mg/cm^2^. The sunscreen was carefully spread across the entire surface by gently rubbing the slide with a nitrile finger cot. Subsequently, the sample was left to dry for 15 min in a dark room before UV transmittance measurements. The UV transmittance of lignin-based sunscreen was measured using a Shimadzu^®^ UV-1900i spectrophotometer. For each lignin-based sunscreen, a minimum of 5 samples were prepared, and measurements were repeated at least 3 times. Transmittance measurements were accumulated in the wavelength spectrum from UVB (290–320 nm) to UVA (320–400 nm). Finally, the *SPF* (Sun protection Factor) value was calculated according to the following equation:

(1) $$SPF=\frac{\sum_{290}^{320}E_{\lambda }S_{\lambda }}{\sum_{290}^{320}E_{\lambda }S_{\lambda }T_{\lambda }}$$

where *E_λ_* denotes CIE erythemal efficiency, *S_λ_* denotes solar irradiance, and *T_λ_* denotes the transmittance of the sample. The values of *E_λ_* and *S_λ_* are constants, and they were determined by Sayre et al. [76].

## 3. Results

The objective of this study was to generate tailored LNPs from various feedstocks through eco-friendly processes and analyze the impact of these parameters on the lignin-based sunscreen’s performance.

### 3.1. LMPs Extraction and Characterization

In order to overcome the current limitations of lignin due to pulping processes, i.e., its extraction using toxic solvents and its relatively high sulfur composition, we purposely used ethanol–organosolv pretreatment without acid catalysis [53]. This method has the advantage of generating clean and pure LMPs with chemical structures that more closely match native lignins compared to kraft ones [53]. The acid-free organosolv process was, therefore, applied to several native biomass types, namely hardwood, softwood, and herbaceous materials. This approach renders it possible to produce lignin fractions with controlled chemical structures, particularly in terms of G, S, and H ratios (Figure 1), which are expected to be crucial for nanometric size reduction and future UV-absorbing properties. The LMPs extraction yields, and physico-chemical features obtained via SEC techniques and NMR (^31^P, and HSQC) are listed in Table 1. It appears that the organosolv process provides advantages by combining very pure lignin production (93.0%, 96.1%, and 93.3% for beech, spruce, and wheat straw, respectively) with high extraction yields (69.7%, 35.9%, and 44.8% for beech, spruce, and wheat straw, respectively, and based on raw lignin biomass content). In addition, the isolation process leads to sulfur-free lignins, while commercial kraft lignin contains up to 1.8% sulfur, which is a key factor for potential product development.

The LMPs molecular weight (Mw) is presented in Table 1 and Table S1. It appears that organosolv delignification treatment led to Mw reduction, which is consistent with lignin breakdown. Furthermore, all three lignin samples extracted using the organosolv process had a lower average Mw (20.8, 15.7, and 17.2 kDa for beech, spruce, and wheat straw, respectively) than the lignin derived from the kraft process (29.4 kDa). These results suggest that both types of feedstock and the extraction process parameters play a part in lignin reaction mechanisms, such as depolymerization and recondensation.

In order to further investigate the chemical structure of different recovered LMPs, ^31^P and two-dimensional HSQC NMR measurements were performed. Table 2 shows the structural assignments and number (mmol.g^−1^ of lignin) of main ^31^P NMR signals for phosphitylated LMPs.

Considering the former ^31^P NMR results obtained from milled wood lignin (MWL) reported by Brosse et al. [77] and Qian et al. [74], a significant increase in phenolic -OH groups was observed due to the β-O-4 aryl ether acidolysis. Kraft lignin appears to undergo higher recondensation according to ^31^P NMR results, with a substantial increase in guaiacyl -OH amounts compared to spruce organosolv lignin. In addition, HSQC NMR analyses were conducted in order to investigate side chains and aromatic region change ratios (β-O-4, β-5, β-β, and S/G amount in %). Milled wood results for the extracted lignin in Table S1 confirmed that organosolv pretreatment led to lignin depolymerization, which was highlighted by β-O-4 acidolytic breakdown. The elevated S/G ratios observed in both beech and wheat straw also indicate lignin depolymerization. Regarding comparative results obtained with organosolv spruce lignin isolation versus kraft lignin, the findings indicate that the kraft process has a more severe influence on chemical structure by promoting depolymerization and recondensation.

It can be concluded that, independently of the feedstock source, organosolv allows the extraction of a lignin type that is close to the native one, with less depolymerization and recondensation compared with the kraft process. Moreover, in accordance with the studies of Adamcyk et al. [53] and Siika-aho et al. [78], delignification and lignin depolymerization are easier for both hardwood and herbaceous materials compared to softwood with respect to the same process severities. Nevertheless, herbaceous material processing challenges still need to be addressed due to their low density and important ash content compared to hardwood and softwood biomass, which makes it necessary to implement specific optimizations for efficient fractionation steps.

### 3.2. LNPs Production and Characterization

**Figure 2:**
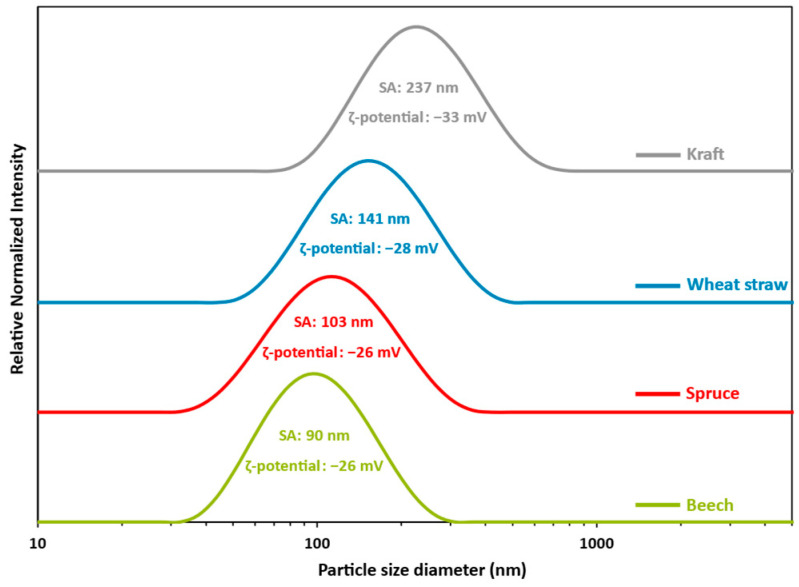
LNPs particles size distributions obtained from a pool of 3 batches, each prepared according to the described protocol: LMPs concentration: 30 mg/mL. Addition rate: 100 mL/min. Anti-solvent composition: 100% water. Dilution ratio: 1/10. T °C: 20 °C. Stirring speed: 500 rpm. SA: size average. PI: polydispersity index. Green: beech; PI = 0.148. Red: spruce; PI = 0.183. Blue: wheat straw; PI = 0.185. Grey: kraft; PI = 0.193.

The size distribution, average size, and ζ-potential of LNPs suspensions obtained according to the manufacturing process described above were analyzed using a Malvern^™^ Zetasizer, as shown in Figure 2. Nanosized, monomodal, and size distributions that are quite large were observed in all cases. The mean size of the particles calculated in terms of scattered intensity decreases in the order kraft > wheat straw > spruce > beech lignin. The mean sizes of particles obtained from beech, spruce, and wheat straw are similar, while particles obtained from kraft lignin are much larger (mean size of 198 nm). The results in Figure 2 support the theory that LNPs formation mechanisms are partly driven by lignin chemical structure interactions [62,79,80,81] since the chemical compositions of lignin molecules extracted from beech, spruce, and wheat straw have more similarities between them than kraft. This highlights the crucial importance of selecting the appropriate biomass for further processing and application. However, the distribution and mean particle size of beech, spruce, and wheat straw do not fall below the limit value of 45 nm defined by Filon et al. [55] with respect to potential skin penetration, providing no restrictions for cosmetic applications. Figure 2 also shows the ζ-potential of the LNPs suspensions. The results revealed a highly negative ζ-potential with respect to all suspensions (−26 to −33 mV), which favors maintaining self-repulsion and electrostatic stability over time. This is confirmed by the very low variation of the particle mean sizes after 90 days of storage at 4 °C (size variation between 3 and 10%).

**Figure 3:**
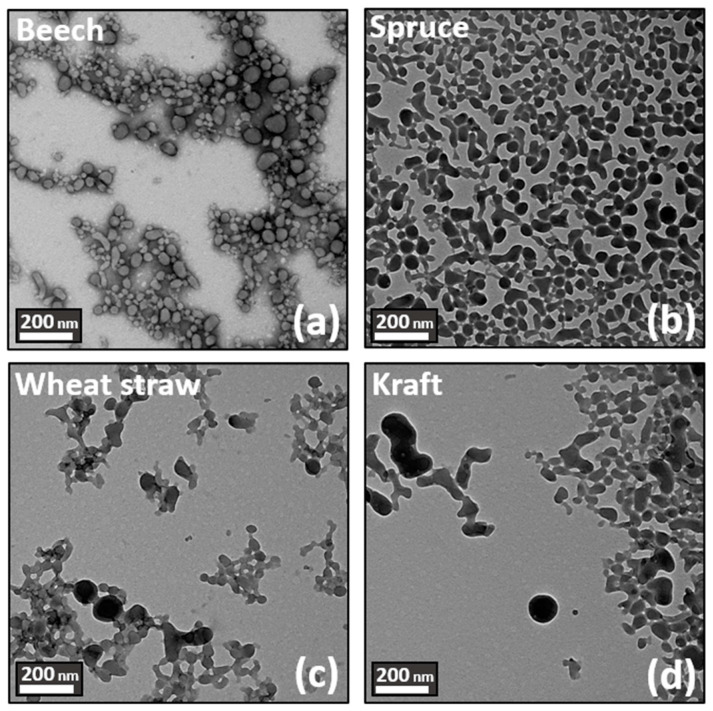
TEM images of the suspension generated according to the fractionation process and biomass source. Top left: (**a**) beech. Top right: (**b**) spruce. Bottom left: (**c**) wheat straw. Bottom right: (**d**) kraft.

TEM microscopic observations in Figure 3 were used to complete the characterization of LNPs. First, TEM images confirm the DLS measurements for each lignin source. Nevertheless, while particles from beech feedstock appeared to be spherical and isotropic, particles from other feedstocks seem to present irregular shapes, as well as anisotropy characterized by a dense core enveloped in a fluffy outer wreath. Finally, it can be observed that Kraft lignin has the most irregular shape and the highest anisotropy.

The multiparameter mechanism behind the formation of lignin nanoparticles in the anti-solvent system can be partially explained by considering the well-documented amphiphilic nature of lignin polymers, as extensively carried out in the literature [61,80,81,82]. Higher phenolic hydroxyl and carboxyl contents enhanced hydrophilicity, while non-covalent π–π interactions contribute to hydrophobicity, especially in lignin with more S units (S > G > H). Consequently, hydrophobic lignin solutions guided by π–π stacking and the S/G ratio yield smaller and more spherical LNPs [83]. Conversely, hydrophilic solutions containing lignins extracted from kraft result in the formation of larger and more irregular nanoparticles due to the presence of hydrophilic components that twist towards the surface of the particle during its formation, thus perturbating the self-assembling process and consequently the aggregation phenomenon (Table 2, Figure 2 and Figure 3) [79].

It can, therefore, be concluded that an optimized LNPs manufacturing method was designed. This procedure allows the obtainment of high production yields and well-defined particle properties (size, shape, stability, and polydispersity) without strongly and negatively impacting the environment from the formulation stage. This is a strong innovative step since, up until now, lignin size reduction had been widely implemented during precipitation with respect to hazardous, toxic, or explosive solvents such as tetrahydrofuran (THF), pyridine, or dimethyl sulfoxide (DMSO) [27].

### 3.3. Lignin-Based Sunscreen Analysis

**Figure 4:**
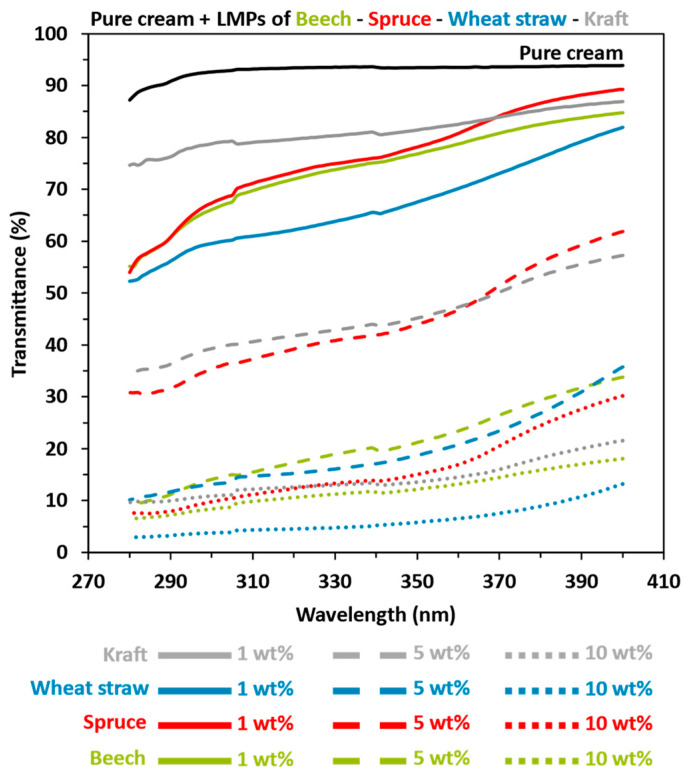
UV transmittance results in the UVA and UVB area of pure cream (NIVEA^®^ Soft) mixed with 1, 5, or 10 wt% lignin macroparticles (LMPs). Black: pure cream. Green: beech. Red: spruce. Blue: wheat straw. Grey: kraft. Solid lines: 1 LMPs wt%. Coarse dotted lines: LMPs 5 wt%. Fine dotted line: LMPs 10 wt%.

For this part, diverse lignin-based sunscreen samples were prepared following the outlined procedure. The investigations focused on assessing the impact of various parameters on UV-absorption sunscreen efficacy, including particle concentration, lignin chemical structure (feedstock and isolation process), and particle size. In each formulation, lignin particles were directly mixed into commercial creams with varying sun protection factors (SPF 1, SPF 10, and SPF 30) to assess the UV-shielding ability of lignin, both with and without other commercial filters. The results were also compared with pure SPF 10 and SPF 30 commercial sunscreens as references. The transmittance and related SPF values of lignin-based sunscreen were measured in the UVA (320–400 nm) and UVB (290–320 nm) ranges, with the results summarized in the following section. First, Figure 4 and Table 3 results indicated that the pure cream (NIVEA^®^ Soft) was UV-filter-free, with a maximum transmittance of 95–90%, corresponding to an SPF of 1.08.

Then, Figure 4 confirms the former results from the literature [19,20,22,24,26,28,75], with a decrease in UV transmittance linked to an increase in SPF values when LMPs were added to pure cream, regardless of the lignin type or biomass feedstock. The SPF values of the 1 wt% LMPs-based pure cream ranged from 1.27 to 1.66, with lower transmittance in both UVA and UVB areas for organosolv–wheat straw lignin (OWS). A substantial gap was observed with respect to 5 wt% of LMPs concerning kraft and organosolv–spruce (OS) compared to organosolv–beech (OB) and wheat straw lignins (SPF values from 2.51 and 7.33). Indeed, the results from 5 wt% LMPs-based OWS and OB pure cream were practically equivalent to kraft and OS 10 wt%. For the 10 wt% amount, OWS stands out from other preparations with an SPF of 25.30 compared to 11.24, 9.65, and 8.85 for OB, OS, and kraft lignin, respectively.

Close trends were therefore observed for hardwood and herbaceous species: transmittance decreases related to UV shielding follow the lignin concentration of lignin-based cream. Conversely, for softwood species, the UV-shielding evolution is more gradational depending on the total amount of lignin inside the cream. The improved results compared to the literature [19,20,22,26] can be explained by the use of organosolv lignin demonstrated by Qian et al. [20], which is most suitable for high-SPF cream. It is also explained by effective homogeneity (i.e., distribution of LMPs in pure cream or commercial sunscreen without aggregates, Figure S1), even with macrolignin compared to the Zhang et al. [75] work.

**Figure 5:**
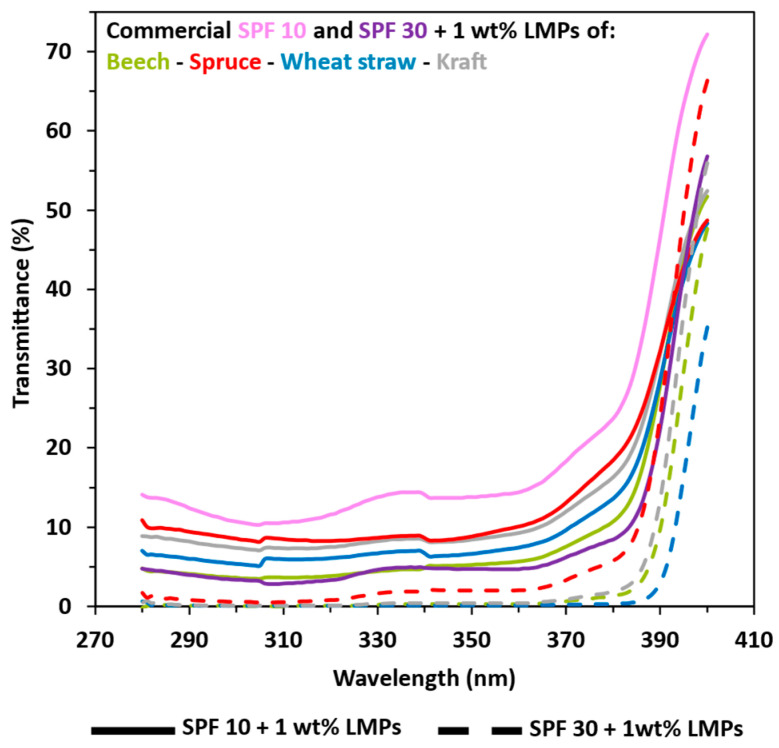
UV transmittance results in UVA and UVB areas from 1 wt% lignin macroparticles (LMPs) blended with SPF 10 (pink) and SPF 30 (purple) commercial sunscreen. Green: beech. Red: spruce. Blue: wheat straw. Grey: kraft. Solid line: commercial SPF 10 sunscreen + LMPs. Coarse dotted line: commercial SPF 30 sunscreen + LMPs. Other results are available in ESI.

Currently, if the brown characteristic color of lignin is still a problem, the solution may lie in the use of this natural organic filter as a complement to current filters in order to reduce their proportions. Therefore, the enhancement of commercial SPF 10 and SPF 30 sunscreens with LMPs was also studied. Results in Figure 5 and Figure S2 indicate that LMPs improve both commercial SPF 10 and SPF 30 sunscreens. The same trends were observed, with hardwood and herbaceous LMPs exhibiting better UV-shielding properties with the transmittance of SPF 10 + 1 wt%, closely matching commercial SPF 30 sunscreens (SPF values of 18.29 and 27.43). For softwood LMPs, even at 5 wt% concentration, the transmittance of SPF 10 corresponds to the SPF 30 sunscreen (SPF values of 22.65 and 26.17). In the case of adding 1 wt% of LMPs in the commercial SPF 30 lotion, the total UV-shielding transmittance value was close to 0 and exhibited highly related SPF values (SPF > 100, but it was indexed at 50+ for a concrete meaning).

**Figure 6:**
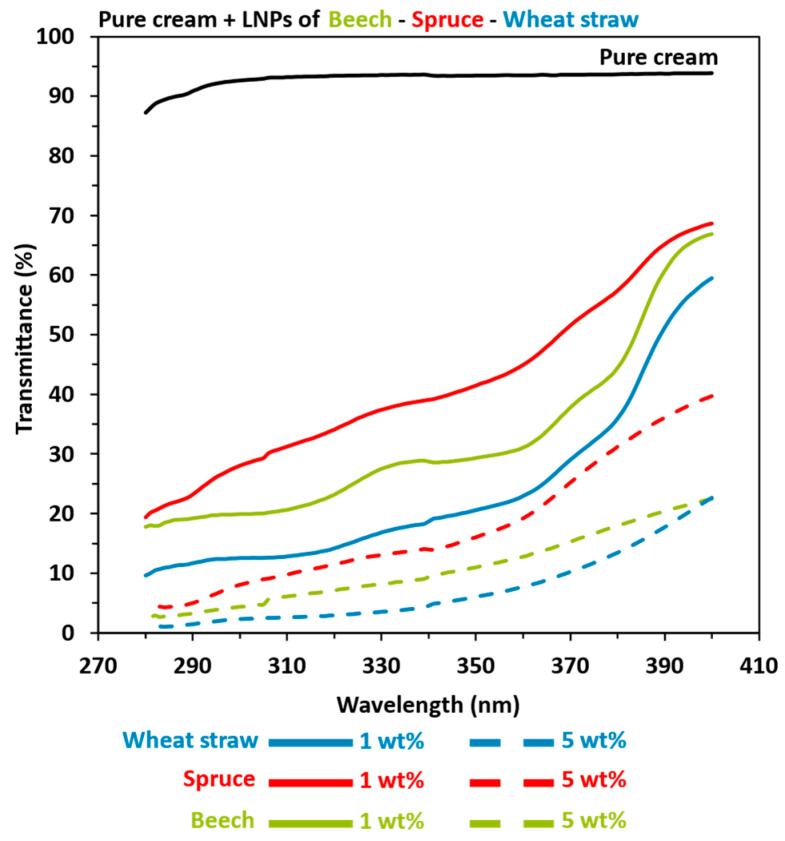
UV transmittance results in UVA and UVB areas of pure cream (NIVEA^®^ Soft) mixed with 1 or 5 wt% lignin nanoparticles (LNPs). Black: pure cream. Green: beech. Red: spruce. Blue: wheat straw. Solid line: 1 LNPs wt%. Coarse dotted line: 5 wt% LNPs.

The enhanced properties observed in a cream blended with lignin can be attributed to two potential factors. First, the chemical composition of the UV filter and its interactions with the constituents of the cream play a crucial role, as was previously demonstrated. Additionally, the size and geometry of the adding filter also contribute to these improvements. Consequently, as shown in Figure 6, we prepared LNPs-based (NIVEA^®^ Soft) on 1 wt% and 5 wt% pure cream from OB, OS, and OWS lignins in order to quantify the reduction size impact over the pure cream properties (UV-shielding and color). Once again, increasing the amount of lignin improved the blended pure cream (NIVEA^®^ Soft) properties, with the following advantage: herbaceous > hardwood > softwood. Indeed, SPF results for the 1 wt% LNP-based pure cream were 7.88, 4.93, and 3.41 for wheat straw, beech, and spruce, respectively. When the creams were prepared with 5 wt% of LNPs, the SPF reached values of 41.97, 20.17, and 11.39 for the same extracted lignin samples.

**Figure 7:**
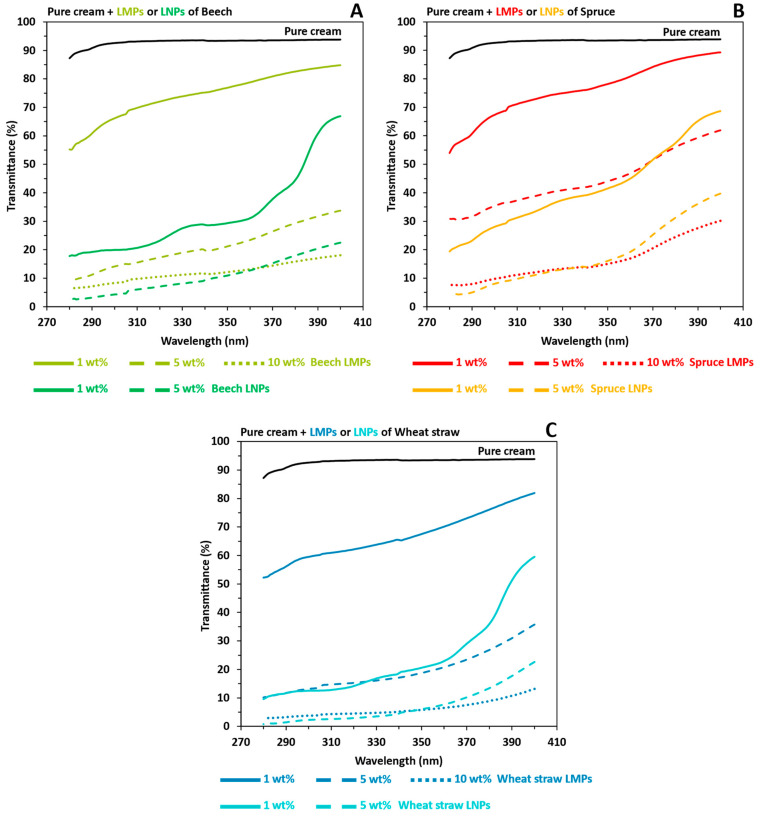
UV transmittance results in the UVA and UVB areas of pure cream (NIVEA^®^ Soft) mixed with the macroparticles (LMPs) or nanoparticles (LNPs) of extracted organosolv lignins. Black: pure cream. (**A**) Beech LMPs or LNPs. Light green: LMPs. Dark green: LNPs. (**B**) Spruce LMPs or LNPs. Red: LMPs. Orange: LNPs. (**C**) Wheat straw LMPs or LNPs. Dark blue: LMPs. Light blue: LNPs. For (**A**–**C**), solid line: 1 LNPs wt%. Coarse dotted line: 5 wt% LNPs.

By comparing UV-shielding properties between prepared LMPs and LNPs pure creams in Figure 7, the lignin particle size impact was demonstrated. Indeed, macro- to nanoscale reductions improved the UVB average transmittance of pure creams prepared with the same lignin wt% from 2.5 to 6.5. Thus, close UVB average transmittance values were not only observed between LMPs 5 wt% and LNPs 1 wt% but also between LMPs 10 wt% and LNPs 5 wt%, which consequently revealed a correspondent brown color reduction for LNPs-based pure cream (Figure S1). Ultimately, sunscreens incorporating 5 wt% LNPs derived from the organosolv extraction achieved SPF values of 20.17, 11.39, and 41.97 for beech, spruce, and wheat straw, respectively, closely approximating those of high-protection lotions with minimal organic UV filter content (Table 3).

### 3.4. Factors Affecting Lignin UV-Shielding Properties

It is generally accepted that there are several factors affecting the UV-blocking performances of lignin polymers. These factors manifest in two different forms according to Zhang et al. [84]: (1) the intrinsic chemical structure of lignin relies on the biomass nature, and (2) the extrinsic factors, including the size, shape, and purity of lignin particles, are determined via extraction and nanoparticle fabrication processes:

(1) Lignin UV-shielding properties are related to chromophore functional groups and especially phenolic hydroxyl groups [22,27,31,75,84]. Guo et al. [85] previously demonstrated that the S phenolic group exhibited stronger UV-absorbing properties due to the additional methoxyl group compared to the G and H units (Figure 1). Consequently, this enhanced the abundance of free electron pairs from oxygen atoms. Part of the results aligns with these explanations, such as beech and wheat straw biomass, which present advantageous chemical structures (high S/G phenolic hydroxyl content, i.e., high methoxy content and darker [24] color) and had greater UV-shielding properties compared to spruce lignin. It was previously demonstrated by Wang et al. [26] that the lignin chemical structure remains unchanged with respect to the nanoprecipitation process, which explains why the same trends can be observed between sunscreens prepared with both LMPs and LNPs. However, the observation that wheat straw lignin exhibited superior, stronger UV-shielding properties over beech lignin suggests that while methoxyl groups and phenolic units may be predominant, they do not fully account for all aspects of lignin-enhanced UV protection. The complex and interconnected structure of wheat straw lignin with proven tricin units [71] and other phenolic end groups, such as aryl acetic acid, can also contribute to the enhanced UV-shielding properties observed in herbaceous lignins [86]. Other chromophores in lignin, such as quinones or -CH=CH-, -C=C-, and -C=O bonds [84] and M_w_ [87], can contribute to UVB and UVA absorbance.
(2) Although the extrinsic properties of lignin are often given less consideration, particle size, shape, and purity can be modified by processes and are also important factors that can explain UV absorption properties. First, the relatively highest level of impurities (7%) was found in beech lignin compared to other organosolv-extracted lignin (3.7 and 3.9% for wheat straw and spruce, respectively), which may reduce UV-shielding properties to a greater extent. In the case of kraft, 9.5% of the impurities coupled with 1.8% sulfur presence can also contribute to lower UV absorption despite a large number of aromatic rings. Regarding lignin particle size and morphology, recent studies [22,88] showed that lignin nanoparticles compared to macroparticles have a higher specific surface area, higher transparency, better dispersibility, and, consequently, better UV-shielding properties per weight (Figure S1). Reduced particle size induces a higher specific surface area, thereby permitting the greater availability of chromophores per unit weight. This results in enhanced UV-shielding properties, lighter coloration, and improved dispersion within the sunscreen formulation and ultimately leads to a reduced reliance on organic UV filter (Figure S1). The results from this study have indeed proven previous observations with respect to a general decrease in UVB average transmittance between 2.5 and 6.5 with the same lignin types but different size scales. With respect to particle shape, Tan et al. [70] showed that the spherical (better A/V ratio) design provided a larger surface area because of the minimum packing density compared with other forms, thus allowing higher chromophore availability and concentration, which can boost UV absorption properties.

## 4. Conclusions

Various sunscreen formulations incorporating the macro- and nanoparticles of lignin biopolymers as a natural UV filter were successfully produced in an ecological and simple manner from different biomass by-products and isolation processes. Consequently, the importance of lignin’s intrinsic (chemical structure) and extrinsic parameters (lignin extraction and LNPs properties) over the UV-shielding properties of sunscreens was highlighted and quantified. First, with respect to chemical structures, grass and hardwoods exhibit a higher prevalence of methoxy groups, resulting in a 2.5 times performance improvement compared to softwoods regardless of particle size and concentration. Then, reducing the particle size from the macro- to nanoscale enhances dispersion, increases specific surface area, and, consequently, amplifies UV absorbance, resulting in a color reduction and improvements between 2.5- and 6.5-fold, irrespective of the lignin’s nature and concentration. From this observation, sunscreen formulated with 5 wt% LNPs from wheat straw achieved an SPF value of 41.97, approaching that of high-protection lotions. When added to commercial sunscreens, lignin boosted performances, while reducing synthetic filter concentrations, providing 50+ protection from SPF 10 lotion with 5 wt% of LMPs. This study provides a simple approach for lignin polymer valorization with the formulation of sunscreens using eco-friendly LNPs.

## Figures and Tables

**Table 1:** LMPs isolation yields and characterization for beech, spruce, wheat straw, and kraft lignin. Mw represents the average molecular weight.

LMPs Characterizations	Organosolv 200 °C 60 min 60/40 EtOH/H_2_O (*v*/*v*)			Kraft
	Beech	Spruce	Wheat Straw	
Extraction yields (wt%)	69.7 ± 0.8	35.9 ± 1.2	44.8 ± 0.4	/
Purity (%)	93.0 ± 0.4	96.1 ± 0.1	96.3 ± 0.7	91.5 ± 0.3
Sulfur content (%)	˂0.05	˂0.05	˂0.05	1.8
Mw (kDa)	20.8	15.7	17.2	29.4

LMPs extraction (wt%) yields are based on raw lignin biomass content. Results include lignin purity (%), sulfur content (%), and average Mw from SEC.

**Table 2:** Physico–chemical analysis of lignin extracts.

^31^P Nuclear Magnetic Resonance (NMR)—Hydroxyl Groups (mmol.g^−1^)				
LMPs	Beech	Spruce	Wheat Straw	Kraft
**Total -OH**	4.19	3.71	2.94	4.62
**Aliphatic -OH**	2.26	1.71	1.27	1.68
**Phenolic -OH**	1.93	1.86	1.44	2.53
Syringyl	1.18	0.14	0.59	0.36
Guaiacyl	0.74	1.59	0.61	2.17
p-Hydroxypenyl	0.01	0.13	0.24	0
**COOH**	0	0.14	0.23	0.41
**Heteronuclear single quantum coherence (HSQC) NMR spectroscopy—linkages (%)**				
**LMPs**	**Beech**	**Spruce**	**Wheat straw**	**Kraft**
**β-O-4 (%)**	25.28	17.89	16.09	9.65
**β-5 (%)**	3.94	13.71	3.94	2.44
**β-β (%)**	6.76	3.06	1.40	3.72
**S/G**	1.87	0	1.03	0

**Table 3:** Measured SPF values for lignin-based pure cream and commercial sunscreens.

LMPs (wt%)	0 ^a^	Beech				Spruce				Wheat Straw				Kraft			
		1	5		10	1	5		10	1	5		10	1	5		10
**Pure cream**	1.08 ± 0.02	1.48 ± 0.03	6.77 ± 1.09		11.24 ± 0.24	1.45 ± 0.05	2.76 ± 0.13		9.65 ± 1.23	1.66 ± 0.02	7.33 ± 1.37		25.30 ± 4.31	1.27 ± 0.02	2.51 ± 0.65		8.85 ± 1.21
**LMPs (wt%)**	**0 ^b^**	**1**		**5**		**1**		**5**		**1**		**5**		**1**		**5**	
* SPF 10	9.39 ± 0.53	27.43		50+		13.70		26.17		18.29		50+		11.88		22.65	
**LMPs (wt%)**	**0 ^c^**	**1**				**1**				**1**				**1**			
* SPF 30	30.93 ± 4.97	50+				50+				50+				50+			
**LNPs (wt%)**	**0 ^a^**	**Beech**					**Spruce**						**Wheat straw**				
		**1**			**5**		**1**			**5**			**1**		**5**		
**Pure cream**	1.08 ± 0.02	4.93 ± 0.53			20.17 ± 2.20		3.41 ± 0.96			11.39 ± 1.32			7.88 ± 0.84		41.97 ± 7.38		

^a^ Commercial pure cream without UV-shielding properties. ^b^ Commercial sunscreen with SPF 10 without lignin addition. ^c^ Commercial sunscreen with SPF 30 and without lignin addition. * Commercial sunscreens.

## Data Availability

The original contributions presented in the study are included in the article/Supplementary Material, further inquiries can be directed to the corresponding author/s.

## Supplementary Materials

The following supporting information can be downloaded at https://www.mdpi.com/article/10.3390/polym16131901/s1. Table S1: Milled wood lignin (MWL) molecular weights and main lignin linkages (%).a Results for untreated wheat straw lignin from Auxenfans et al. [89].; Figure S1: Appearance of each produced sunscreen; Figure S2: UV transmittance of commercial SPF 10 sunscreen mixed with 5 wt% LMPs from organosolv (beech, spruce, and wheat straw) or kraft (spruce) processes in UVA and UVB areas.
