# Supplementary material for: The Impact of Lignin Biopolymer Sources, Isolation, and Size Reduction from the Macro- to Nanoscale on the Performances of Next-Generation Sunscreen
Source: Polymers (Basel). 2024 Jul 2;16(13):1901. doi: 10.3390/polym16131901 (PMC11244244; doi:10.3390/polym16131901)
Supplement: Supplementary file 1 [file polymers-16-01901-s001.zip › polymers-3057901-supplementary.pdf]

Supplementary materials for the article:

The Impact of Lignin Biopolymer Sources, Isolation, and Size Reduction from the Macro- to Nanoscale on the Performances of Next-Generation Sunscreen

Victor Girard [1,*], Léane Fragnières [1], Hubert Chapuis [1], Nicolas Brosse [1], Laurent Marchal-Heussler [2], Nadia Canilho [3], Stéphane Parant [3] and Isabelle Ziegler-Devin [1]

[1] Laboratoire d'Etude et de Recherche sur le MAtériau Bois (LERMAB), Faculty of Science and Technology, University of Lorraine, F-54000 Nancy, France; leane.fragnieres@etu.unistra.fr (L.F.); hubert.chapuis@univ-lorraine.fr (H.C.); nicolas.brosse@univ-lorraine.fr (N.B.); isabelle.ziegler@univ-lorraine.fr (I.Z.-D.)
[2] Ecole Nationale Supérieure des Industries Chimique (ENSIC), University of Lorraine, F-54000 Nancy, France; laurent.marchal-heussler@univ-lorraine.fr
[3] Laboratoire Lorrain de Chimie Moléculaire (L2CM), Faculty of Science and Technology, University of Lorraine, F-54000 Nancy, France; nadia.canilho@univ-lorraine.fr (N.C.); stephane.parant@univ-lorraine.fr (S.P.)
* Correspondence: victor.girard@univ-lorraine.fr

Table S1: Milled wood lignin (MWL) molecular weights and main lignin linkages (%).

Figure S1: Appearance of each produced sunscreen.

Figure S2: UV transmittance of commercial SPF 10 sunscreen mixed with 5 wt% LMPs from organosolv (beech, spruce, and wheat straw) or Kraft (spruce) processes in UVA and UVB areas.

Table S1: Milled wood lignin (MWL) molecular weights and main lignin linkages (%).[a] Results for untreated wheat straw lignin from Auxenfans et al. [1].

Milled Wood Lignin (MWL) characterization results			
	Beech	Spruce	Wheat Straw[a]
Mw (kDa)	65.7	36.9	-
β-O-4 (%)	47.94	42.79	45.30
β-5 (%)	3.93	11.70	8.30
β-β (%)	12.03	3.49	5.60
S/G	0.57	0	1.12

Results includes average molecular Mw from SEC and quantification of side chains (β-O-4, β-5, β-β) from HSQC NMR spectra.

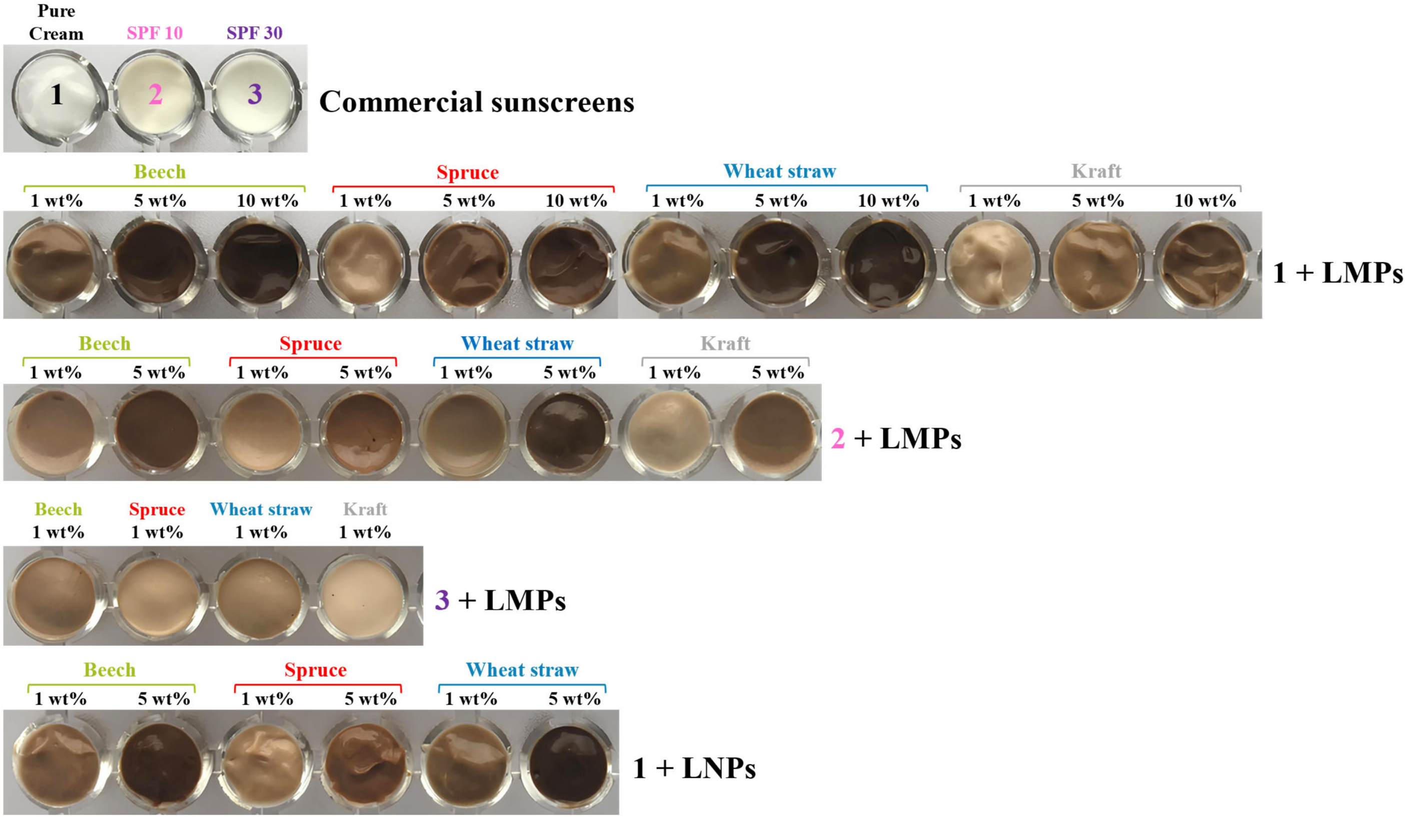

Figure S1: Appearance of each produced sunscreen. First line: the three commercial cream and sunscreen (1: moisturizing Nivea soft cream with SPF 1 labelled as "pure cream"). Second line: Pure cream blended with the lignin macroparticles for the three organosolv lignin or the kraft commercial at 1, 5 and 10 wt%. Third line: SPF 10 commercial sunscreen mixed with the four lignins at 1 and 5 wt%. Fourth line: SPF 30 commercial sunscreen blended with the four lignins at 1 wt%. Fifth line: Pure cream blended with the lignin nanoparticles for the three organosolv lignin at 1 and 5 wt%.

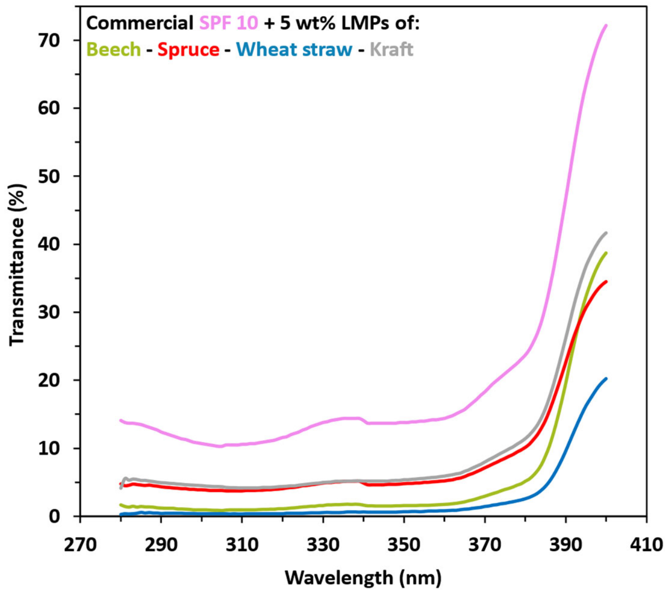

Figure S2: UV transmittance of commercial SPF 10 sunscreen mixed with **5 wt% LMPs** from organosolv (**beech**, **spruce**, **wheat straw**) or Kraft (from spruce) processes in UVA and UVB areas.

Reference :

1. Auxenfans, T.; Crônier, D.; Chabbert, B.; Paës, G. Understanding the Structural and Chemical Changes of Plant Biomass Following Steam Explosion Pretreatment. *Biotechnol Biofuels* **2017**, *10*, 36, doi:10.1186/s13068-017-0718-z.
